# Feedback and efficient behavior

**DOI:** 10.1371/journal.pone.0175738

**Published:** 2017-04-21

**Authors:** Sandro Casal, Nives DellaValle, Luigi Mittone, Ivan Soraperra

**Affiliations:** 1 Department of Economics, Management, and Quantitative Methods, University of Milan, Milan, Italy; 2 School of Social Sciences, University of Trento, Trento, Italy; 3 Department of Economics, University of Trento, Trento, Italy; 4 Department of Economics, University of Amsterdam, Amsterdam, Netherlands; Technion Israel Institute of Technology, ISRAEL

## Abstract

Feedback is an effective tool for promoting efficient behavior: it enhances individuals’ awareness of choice consequences in complex settings. Our study aims to isolate the mechanisms underlying the effects of feedback on achieving efficient behavior in a controlled environment. We design a laboratory experiment in which individuals are not aware of the consequences of different alternatives and, thus, cannot easily identify the efficient ones. We introduce feedback as a mechanism to enhance the awareness of consequences and to stimulate exploration and search for efficient alternatives. We assess the efficacy of three different types of intervention: provision of social information, manipulation of the frequency, and framing of feedback. We find that feedback is most effective when it is framed in terms of losses, that it reduces efficiency when it includes information about inefficient peers’ behavior, and that a lower frequency of feedback does not disrupt efficiency. By quantifying the effect of different types of feedback, our study suggests useful insights for policymakers.

## Introduction

A robust finding in economics, psychology, and behavioral sciences is the systematic failure to act according to rational well-informed preferences [1]. This failure to rationally process and integrate information due to limited cognitive resources may lead to inefficient behavior in many domains of everyday life and may produce costs that, in some cases, can be avoided simply by highlighting the consequences of such behavior.

Individuals make a huge number of choices every day that, in most cases, are implicit and can lead to suboptimal outcomes. Potential reasons might be habit formation and unawareness of the consequences of alternative courses of action. A prominent example of unawareness of the consequences of alternative courses of action are savings and investment decisions. In retirement saving decisions, for instance, investors are relatively passive and do not spend time collecting information. They often abstain from joining advantageous plans, they tend to stick to the default option and do not explore new alternatives that may lead to more efficient choices [2]. Suboptimal behaviors may lead actual workers to not save enough, which may result in a generation of future poor pensioners. For this reason, regulators and policy makers have implemented strategies for sustaining workers’ investment into retirement plans. Often these strategies include automatic enrolment [3] or the information about about peer behavior on savings. With respect to the latter, Duflo and Saez (2000, 2003) [4] [5] found that it is effective at increasing individual likelihood to enrol into a retirement saving plan. Whereas, more recently, Beshears *et al.* (2015) [6] found that it has a negative impact on retirement saving rates of those who are not saving enough.

Another example of suboptimal decisions due to a lack of awareness of the consequences of alternative courses of action is domestic electricity consumption. Electricity consumption is one of the most debated cases in which habits lead to individually and socially inefficient decisions. In this case, alternative courses of action are not salient to the consumer who undertakes consumption without well-informed preferences. Indeed, when individuals consume a given amount of KWh by using the most disparate appliances, they are certainly better off but, at the same time, they may fail to properly assess the costs and benefits associated with their choices and with alternative behaviors. The difficulty to assess choice consequences for this type of good lies in the fact that consumers are not directly concerned about consuming electricity that, as Fischer (2008) [7] points out, is invisible and partially outside their control. Real attempts of driving electricity consumers towards a virtuous behavior are those implemented by some companies which, together with the electricity bill, send a detailed report in which the consumption of the household is compared to the one of the neighbors (see [8] for a discussion of a real life situation). Unfortunately, also in this real example, results on how individuals react to received information about peers’ behavior (in this case on energy consumption) are not clear-cut (see, for instance, [9] and [8] on the so called *boomerang effect*).

Unawareness of consequences may also lead to poor choices not only in saving or consumption but also in other domains. For example, cars nudge drivers when they have to change gear, insert the neutral gear or use the clutch pedal. Advanced vehicle computers keep track of drivers’ performances and summarize how many kilograms of *CO*_2_ (or litres of gas) the driver saved. Without these mechanisms, drivers would be satisfied with their suboptimal behavior, ignoring the existence of a better driving style which is implementable through small changes.

In all these settings, the decision-makers’ inability to make efficient choices plays a crucial role. As already pointed out, the lack of awareness of the consequences of alternative courses of action may lead to individuals being more exposed to cognitive biases, and individuals may settle for satisfying (but inefficient) behavior. By stopping the search for better alternatives, they might lose the opportunity to join an advantageous pension plan, to gain additional benefits from changing their driving style or to reduce additional losses from efficiently consuming electricity.

A powerful and affordable strategy that can enhance awareness of choice consequences and foster the search for efficient alternatives is feedback. Feedback enables us to fill a “knowledge gap” that individuals face when they cannot access the level and the rate of their behavior, thus, it stimulates the exploration of behavioral alternatives and search for the most efficient one [10].

In this study, we enrich the literature on feedback and efficiency by providing laboratory evidence on the mechanisms underlying the feedback effects on efficient behavior. In particular, we investigate how different types of feedback, including social information and framing effects, enhances awareness of behavior consequences and stimulates exploration.

## Related literature

Research on the effectiveness of feedback on behavior change has a long history in psychology and behavioral economics. By providing individuals with information about the consequences of their past behavior, feedback represents a powerful strategy for enhancing learning and better performance [11, 12].

In the consumer research literature, the role of feedback has mainly been investigated in terms of the effects of knowledge of results [13–15]. Providing information about past performance enhances individuals’ learning of consequences and, thus, better consumption decisions [16–18].

In the literature on learning, feedback is identified as a mechanism that causes a change in behavior such that decisions might converge to efficiency. This line of research has typically employed laboratory experiments in which subjects face the same task repeatedly without prior information about options’ payoff distribution and receive a feedback after each choice [19]. This task is usually a choice under uncertainty between (at least) two options in the so-called *clicking paradigm*. Often these experiments involve *small feedback-based decisions*, which are characterized by i.) repeated decisions, ii.) small importance of each single decision, and iii.) unawareness of a prior information concerning the payoff distributions (see, for instance, [20]). Learning research evidence suggests that the effect of experience on choice behavior depends on the type of feedback received, with different types of feedback leading to different processes [21]. In particular, the effect of feedback about obtained payoff is captured by reinforcement learning models: agents are assumed to evaluate their choices by looking only at obtained payoffs. While the effect of feedback about foregone payoffs, i.e., the payoff that could have been received by selecting another action, is captured by beliefs-based learning models: agents are also assumed to incorporate foregone payoffs in the learning process [22]. Some evidence supports beliefs-based learning models: when feedback contains also foregone payoff, the probability of re-evaluating disappointing options increases [21]. This positive effect on learning is observed especially when the decision-making context has low variance, there are several alternatives, and payoffs associated with the different options are positively correlated [22].

Despite this evidence, in many natural settings individuals receive a feedback limited to the payoff obtained by selecting an option, as depicted by reinforcement learning models. In these settings, individuals have to explore to learn the incentive scheme by looking at obtained payoff.

Laboratory evidence from the learning research field suggests that a change in feedback about obtained payoff may enhance learning and better performance. In particular, when the *frequency* of feedback about obtained payoff is high, it has a positive effect on learning and performance [23, 24]. Similarly, the literature on reinforcement learning suggests that when the frequency of feedback is reduced (partial reinforcement), it has a negative effect during acquisition—i.e., in the learning phase when behavior is reinforced—and a positive effect in the extinction phase—i.e., when the behavior is not reinforced anymore [25]. This effect, which is known as the partial-reinforcement extinction effect, suggests that even though learning is slower, learned behavior is more robust under partial reinforcement compared to continuous reinforcement. Despite this evidence, it is not clear whether a higher frequency of feedback has a positive effect on learning. In particular, Lam *et al.* (2011) [26] show that when feedback is too frequent it has a negative effect on learning and performance in the early phases of learning. This effect can be related to information overload: as feedback frequency increases, individuals use available cognitive resources to process feedback information instead of using them to learn the task.

Feedback containing others’ obtained payoff (which can also introduce foregone payoffs) might also have a positive effect on learning and efficiency [27]. Social learning literature suggests that agents draw inference from others’ choices in settings characterized by uncertainty [28]. In these settings, providing feedback about their own and others’ obtained payoff invokes a *social comparison* that elicits a positive behavioral change [29, 30].

Finally, there is evidence that when feedback includes *losses* it has a positive effect on learning and efficiency. This effect has been attributed to the phenomenon of loss aversion [31], i.e., under loss aversion individuals assign higher subjective weight to losses than to gains [32], and also to the fact that losses stimulate task attention [33]. In this regard, the positive effect of losses on performance has been questioned in artificial settings. For instance, [34] is a replication of the field study of Ganzach and Karsahi (2005) [35] aimed at validating the efficacy of losses on efficient credit card use also in artificial environments. The authors do not find that losses have a positive effect on behavior, advocating that in artificial environments subjects are less involved than in natural settings and, thus, are less willing to process information optimally. Although our experiment is an artificial environment, our subjects are guaranteed to be highly involved by taking part in incentivized tasks (see *Induced Value Theory* [36]).

Several field studies have employed feedback as a mechanism to enhance efficiency in the field of energy consumption [7, 37–41]. Other studies [8, 9, 42] tested the effect of *social feedback* on energy conservation and suggest that it is effective at increasing energy conservation. On the other hand, a review of several studies published in the psychological literature [7] shows that feedback containing social information elicits a mixed behavior change. To explain this inefficacy, the author of the review refers to the boomerang effect: suboptimal consumers might be motivated to change their consumption behavior toward the efficient one, while efficient consumers might be exposed to the risk of worsening their behavior. As for the frequency of feedback, there are studies suggesting that *frequency* has a positive effect on energy consumption: the more frequent the feedback is, the higher the effect on learning choice consequences [7, 43, 44]. The same studies [7, 44] suggest that *framing* is also crucial for stimulating exploration and fosters search for better consumption alternatives. Conditional on how information is framed, feedback can activate different motivations and, thus, behavior changes. In particular, recent evidence shows that disclosing information about losses associated with consumption motivates energy conservation [40].

The effect of feedback on energy efficiency has also been investigated in the laboratory by simulating consumer behavior in a virtual home by asking subjects to consume energy by using seven different groups of items [45]. In this paper, energy consumption is investigated along five dimensions in which information changes. In the baseline, participants were informed about the net gain associated with their consumption choices on an invoice screen. Depending on the treatment, either they received some advice about energy saving before the choice was made through a smart meter display or they received the average and minimum energy consumption level in their market. Although this recent contribution, the effect of feedback on energy efficiency remains under-investigated in controlled environments.

Our study adds to the research on feedback and efficiency by providing laboratory evidence on the mechanisms underlying its effects on behavior. We simulate the problem faced by an individual who has limited awareness of the consequences associated with her choices. We introduce feedback about obtained payoffs as a mechanism to enhance awareness of choice consequences and the learning of better alternatives. We manipulate the social content, the frequency, and the frame of feedback about obtained payoffs along six dimensions.

In addition, we add to the methodology of the learning research field by introducing a novel task. Experimental subjects are provided with a task in which they have to choose how to allocate experimental points to five items that convert these points into a final payoff according to a function that is unknown to the subjects. As in the case of a decision-maker who has to decide how to use her resources with limited knowledge of the consequences of different courses of action—e.g., an investor that needs to decide how to invest her capital—our subjects do not know how items convert points in the payoff but they have the opportunity to find more efficient combinations through exploration.

Our study also adds to the research on learning and feedback by highlighting two mechanisms underlying feedback efficacy on learning and efficiency. The first finding relates to the fact that feedback including losses elicits a substantial positive effect on learning and fosters search for efficient alternatives. The effect of losses on performance is still underinvestigated in the learning research and, thus, our result represents an original contribution to this literature. The second finding adds to the social learning research and the controversial evidence on the efficacy of social feedback. When feedback includes social information about an inefficient best performer, it elicits a negative effect on learning and exploration.

Finally, our study provides useful insights for policymakers by quantifying the effect of different types of feedback on efficiency.

## Materials and methods

We design an experiment that mimics the problem faced by an individual who has limited awareness of the consequences of her choices and potentially forgoes additional benefits by undertaking poor behavior. Our aim is to provide laboratory evidence on the mechanisms underlying the feedback effects on the search for better alternatives and awareness of choice consequences by manipulating three dimensions of feedback: *framing*, *frequency of delivery*, and *social content*.

We investigate individual behavior as performance in a repeated *allocation task* of points to various items: experimental subjects are asked to simultaneously use five sliders by choosing the amount of points to allocate to each. Participants cannot assess the benefits and costs associated with their allocation choices. Therefore, we introduce feedback as a way to enhance awareness of choice consequences and to foster the search for more rewarding allocations. Participants are told that only one allocation is efficient, i.e. is the most rewarding.

Participants earned the points they are asked to allocate to the sliders through an *effort task*. This task controls for the effect of experimental asset origin. While windfall assets have been associated with several behavioral anomalies, asset earned through effort has been associated with higher self-interested behavior [46]. The effort task was based on [47]. Participants had 50 minutes to correctly count the number of zeros in 21 different tables with 150 randomly ordered zeros and ones. In our experiment, participants earned 50 points for each correctly solved table. Moreover, they were informed that they could proceed to the allocation task only upon the successful completion of all the 21 tables in the effort task. All participants managed to complete the 21 tables and, therefore, at the end of the effort task, they earned 1,050 points. Individual cognitive ability might confound performance in the allocation task. Therefore, to determine a measure of individual ability, we recorded the time individuals needed to complete the effort task.

In the *allocation task* participants were asked to allocate to five different sliders *j* ∈ {1, …, 5} the points (1,050) they had earned from completing the effort task. They were told the task would be repeated for 21 rounds. In each round, participants had to allocate 50 of the 1,050 points by deciding the number of points *x*_*j*_ ∈ {0, …, 20} to assign to the j-th slider. Participants were required to use all the available points and were allowed to allocate a maximum of 20 points to a single slider.

The first dimension of feedback that we manipulated is the *framing* of information: when *framing* was positive, feedback included obtained payoff as benefits: the j-th slider generated a payoff *π*_*j*_(*x*_*j*_) that depended on the number of points *x*_*j*_ allocated to that slider according to the function

$$\begin{array}{c} \pi_{j}\left(x_{j}\right)=c_{j}\exp\left(-\frac{{\left(x_{j}-m_{j}\right)}^{2}}{2s_{j}^{2}}\right) \end{array}$$

where:

- *c*_*j*_ determines the slider’s maximum payoff,
- *m*_*j*_ determines the number of points required for the slider’s maximum payoff and
- *s*_*j*_ determines how fast the payoff decreases by moving away from the maximum.

**Fig 1:**
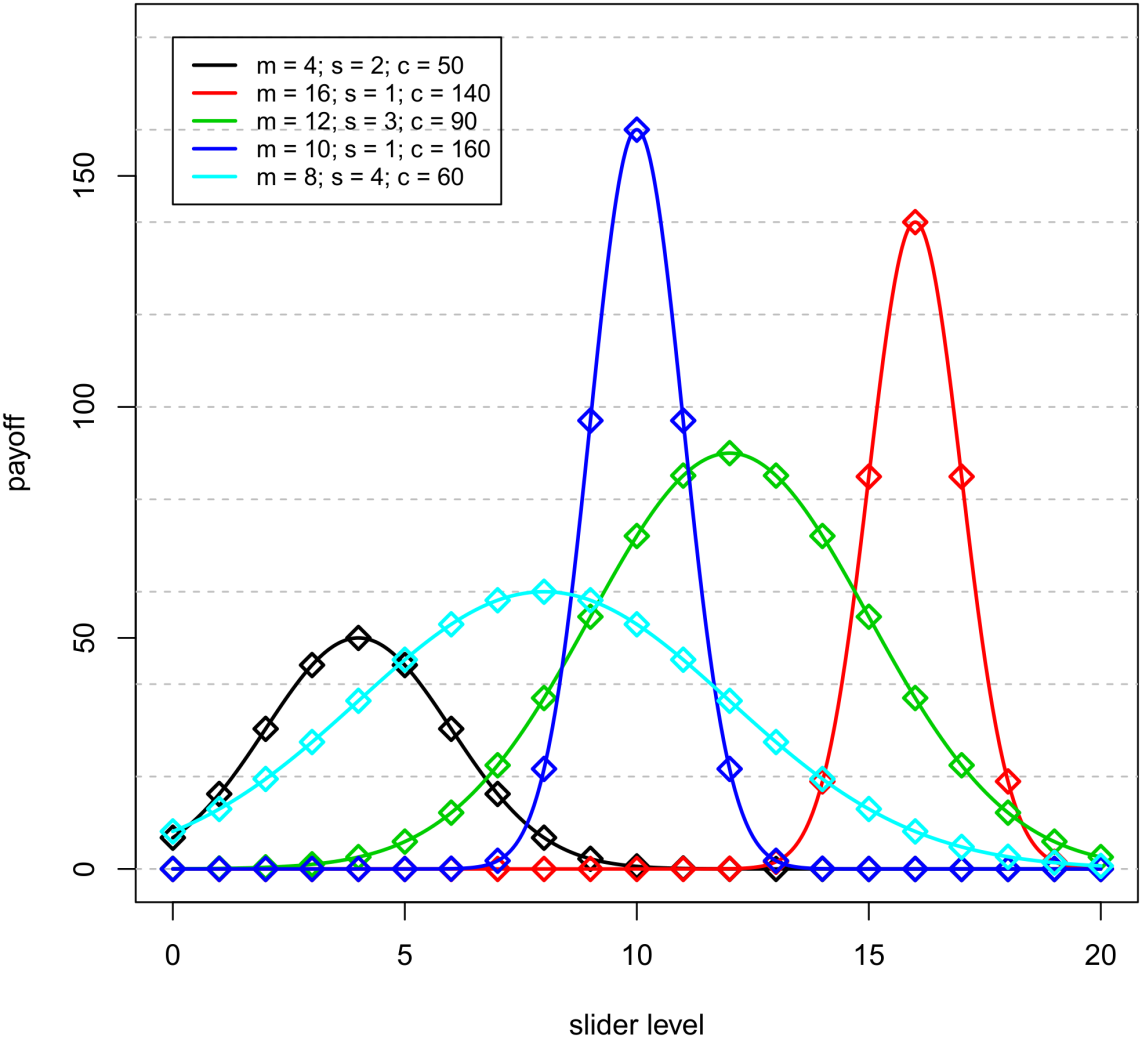
Sliders’ payoff functions.

The total payoff for the round was then computed as $\Pi \left(x_{1},\ldots ,x_{5}\right)=\sum_{j=1}^{5}\pi_{j}\left(x_{j}\right)$. Fig 1 shows the payoff functions and the parameters characterizing each slider.

Each slider’s payoff function was unknown to the participants, they only knew the minimum and maximum attainable total payoffs (0 and 500 ECU, respectively).

When *framing* was negative, participants received a feedback including the obtained payoff in terms of *losses*. This treatment allows us to assess the effect of losses on behavior: losses increase task attention [33] and loom larger than same-sized gains [32]. In this treatment, the j-th slider generated a loss (*C*_*j*_(*x*_*j*_)) that depended on the number of points *x*_*j*_ allocated to that slider according to the function

$$\begin{array}{c} C_{j}\left(x_{j}\right)=c_{j}\left(1-\exp\left(-\frac{{\left(x_{j}-m_{j}\right)}^{2}}{2s_{j}^{2}}\right)\right) \end{array}$$

where:

- *c*_*j*_ determines the slider’s maximum loss,
- *m*_*j*_ determines the number of points required for the slider’s minimum loss (zero for all sliders) and
- *s*_*j*_ determines how fast the loss decreases by moving away from the maximum.

**Fig 2:**
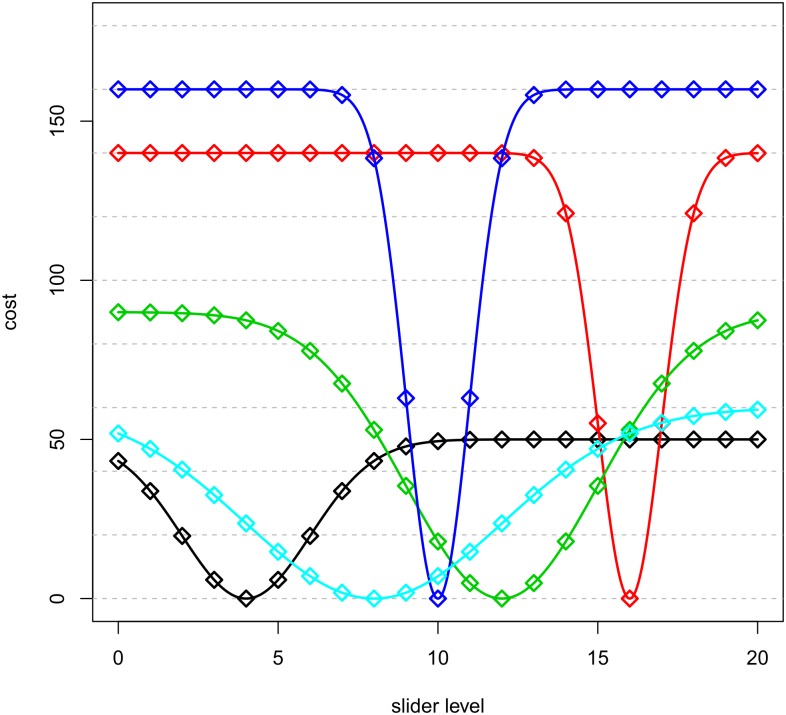
Sliders’ cost functions.

Obviously, the loss of each allocation is computed to keep the final payoff unchanged across frames: Fig 2 shows the loss functions and the parameters characterizing each slider in the negative framing. It is important to notice that when a slider generates a payoff of *p* in the gain domain it generates exactly a loss of *c*_*j*_ − *p* in the loss domain. To maintain the opportunity of performance comparisons in different frames, at the beginning of each round participants were endowed with 500 ECU and were informed of the losses associated with the chosen allocation. The payoff was determined by subtracting the loss of the allocation from the endowment of 500 ECU.

The second dimension of feedback that we manipulated is *frequency*. Participants received information on the obtained payoffs either at the end of each round or every three rounds (thus, only at the end of round 3, 6, 9, 12, 15, 18, and 21). The main difference is the quantity of feedback received by each subject. Indeed, while the quality of feedback does not differ among conditions, the quantity of feedback differs in that participants received feedback on their past performance 21 times under the first frequency condition, and seven times under the second one. Importantly, when participants received information every three rounds, they were informed about the payoffs obtained from all the previous three rounds. This way, at period 3, 6, 9, 12, 15, 18, and 21 the amount of information available to the participants was the same across all conditions.

The rationale for this manipulation is to explore whether aggregating the feedback over multiple rounds is harmful for efficiency. On the one hand, continuous feedback might lead to higher efficiency. On the other, continuous feedback provision might lead to information overload and can be, very often, infeasible or unsustainable (e.g., for budget or technical constrains). Our manipulation allows us to shed light on the effect of information (dis)aggregation on efficient performance. If diluted feedback has the same effect of continuous feedback then this insight can be leveraged by policymakers, especially when continuous feedback provision is costly. If diluted feedback does not harm individuals’ performances, it is possible to induce the same level of efficiency at a lower cost by reducing the frequency of feedback.

By combining *framing* and *frequency* in a 2x2 design we obtain four experimental treatments.

- In Treatment *straight*-×1, the sliders generate payoffs and the participants receive feedback every round.
- In Treatment *straight*-×3, the sliders generate payoffs and the participants receive feedback every three rounds.
- In Treatment *reverse*-×1, the sliders generate losses and the participants receive feedback every round.
- In Treatment *reverse*-×3, the sliders generate losses and the participants receive feedback every three rounds.

We consider as our Baseline the Treatment *straight*-×1, thus the impact of our manipulations on feedback can be detected by comparing *straight*-×1 with *straight*-×3 (for the *frequency* dimension) and *straight*-×1 with *reverse*-×1 (for the *framing* dimension).

As a third experimental dimension, we manipulated the content of feedback by including *social information*. Treatments on social content followed the basic structure of the baseline *straight*-×1. In addition, we provided participants with information not only about their own payoff and allocation choices in each round, but also with information about the payoff obtained by another subject in each round to address a still open question in the literature. In fact, experiments on imitation in learning from experience do not always find robust results. Some works conclude that the learning process is faster in subjects that may observe others’ performances [48], others claim that personal experience triggers learning better than observed experience [49]. In a comprehensive review, Alos-Ferrer and Schlag (2009) [50] support the role of imitation in fostering efficient behavior. Studies on energy consumption [8, 9] and saving behaviors [5, 6] do not converge to a clear conclusion on the effect of social feedback on performance. Therefore, the effect of disclosing a target individual’s performance on observers’ behavior needs to be clearly addressed.

Our social feedback treatments are *info-eff* and *info-ineff*. In both treatments, participants received feedback about the payoff obtained by the *best-performer* of a group of subjects that participated in a pilot session of the baseline *straight*-×1. The pilot session was conducted to collect data on best-performers. In this session, participants were arranged into groups of five or six. In addition to receiving information about individual obtained payoffs, participants were informed of the payoff obtained by the best-performer of their group. For the sake of completeness, we compared performance in treatment *straight*-×1 and in the pilot treatment without finding, on aggregate, any significant differences. This is not surprising given that different types of best-performers emerged from each group. The effects of providing heterogeneous social feedback cancel out each other.

The *info-eff* and *info-ineff* treatments differ in the best-performer’s category: in *info-eff* participants were informed about the payoff obtained by an efficient best-performer, i.e., the best performer of a group in which participants reached the efficient allocation; while in the *info-ineff* participants were informed about the payoff obtained by an inefficient best-performer, i.e., the best-performer of a group in which none reached the efficient allocation. Importantly, participants in the *info-eff* and *info-ineff* were not informed of the best-performer’s category (e.g., efficient or inefficient).

With these two additional treatments we want to tackle the effect of feedback embedding *social information*, not only by testing whether observing the performance of an individual considered among the best players sets the performances of the observers to higher levels, but also by controlling whether the best performance represents a sort of benchmark which, once reached, prevents exploration. Indeed, if information on the obtained payoff of a best-performer creates a reference point for the observers, setting this reference point to high (*info-eff*) or low (*info-ineff*) levels may influence observers’ behaviors and performances. By construction, the main difference between the *info-eff* and *info-ineff* treatments is only the level at which the reference point is set (high or low). Thus, participants in the *info-eff* treatment do not receive a *better* feedback (feedback does not contain information on how the best-performer reached that specific result), but they are exposed to a higher reference point.

Table 1 summarizes our experimental treatments. All treatments were run in a between-subjects design. To provide transparent incentives for the revelation of truthful behavior in each round, one round was randomly selected to be paid out at the end of the experiment.

**Table 1:** Experimental treatments.

*Treatment*	*Frame*	*Frequency*	*Social content*
straight-×1	Positive	every round	none
straight-×3	Positive	every 3 rounds	none
reverse-×1	Negative	every round	none
reverse-×3	Negative	every 3 rounds	none
info-eff	Positive	every round	efficient best-performer
info-ineff	Positive	every round	inefficient best-performer

### Participants and procedures

The experiment was run at CEEL (Cognitive and Experimental Economic Laboratory) of the University of Trento (Italy). Participants were students of the same university recruited among the members of the laboratory database. The laboratory database contains only the entries of those subjects who indicated their consent—those who signed the appropriate informed consent paperwork—to voluntarily participate in experimental research in the domain of social sciences. Thus, this experiment does not involve people unable to give informed consent, vulnerable individuals or minors.

As anticipated, participants knew that the research was in the domain of social sciences and did not have any medical purpose or content. At the same time, they were aware that the data collected would be treated anonymously and would be analyzed on aggregate without the individual data being traced back to the originator. In addition, we informed participants that sensitive personal data or genetic information would not be collected.

Participants were paid a participation fee (show-up fee) and a fee proportional to their effort and results in the experiment, and this was made clear before the start of the experimental session. Within the experimental economics community experiments that fulfill these requirements are usually conducted without an explicit IRB review. This is also the case of this specific study.

Each experimental session was conducted on two subsequent days: the recruitment message informed participants that they had to guarantee their availability on both days. The first day was dedicated to the effort task (solved, on average, in 27.76 minutes) and the second to the allocation task (one session lasted 60 minutes on average). Before starting the experiment, participants received detailed instructions on the experimental procedure (see S1 File for a translated version of the instructions). As previously mentioned, participants were ensured a fair earning from participating in the experiment: in addition to a show-up fee of 4 Euros, participants received the result of one randomly selected round of the allocation task. The exchange rate was 25 ECU = 1 Euro. On average, individual total earnings amounted to 16.21 Euros.

The experiment was conducted using both the z-Tree software [51] (used for the effort task) and a software developed at the laboratory (for the allocation task). In total, 162 participants took part in the experiment (if we also include the participants to the pilot, we have a total of 209).

## Results

In order to investigate how different types of feedback about obtained payoff enhance individuals’ awareness of choice consequences and the search for better alternatives, we consider two different measures of performance: (i) the round in which subjects reach the efficient allocation, and (ii) the fraction of rounds in which subjects make efficient allocation choices.

There would be a third natural candidate measure of performance: the payoff obtained by the subjects in the experiment. However, in our design payoffs are bounded between 0 and 500 ECU. Since many subjects hit the upper bound, this measure would not allow us to properly identify differences in performance among treatments. Indeed, performances tend to converge at the end of the experiment when subjects progressively reach the efficient payoff. A workaround to the problem is to employ a censored regression model to identify the effects. However, this strategy implies that the dependent variable is censored and can theoretically assume values above the threshold. This is not the case in our setting. Payoffs higher than the efficient one have no meaningful interpretation and cannot be obtained. For this reason, we provide only descriptive information on the payoff dynamic in the main text and test treatment effects using the measures of performance mentioned above. For the sake of completeness and transparency we report in Table A in S1 File the results of random effect censored regression models where payoffs are used as dependent variable.

To analyze the first measure of efficient choices, i.e., the round in which subjects reach the efficient allocation, we use a duration model, whereas to analyze the second measure, i.e., the fraction of efficient choices, we employ a fractional response model. Compared to the OLS regression with logit transformed data, the fractional response model has the advantage of naturally allowing for observations with values of 0 and 1. For details about these models see [52] and [53].

In the following, we describe the results with the aim to isolate and quantify the effect of the three dimensions of feedback (*framing*, *frequency*, and *social content*) we manipulated in our six treatments. In particular, we first discuss the impact of framing effects and the frequency of feedback and we then move to the effect of social feedback.

### Framing effects and frequency of feedback

In this subsection, we consider behavior in the treatments *straight* and *reverse* with feedback every round (×1) and every three rounds (×3).

**Fig 3:**
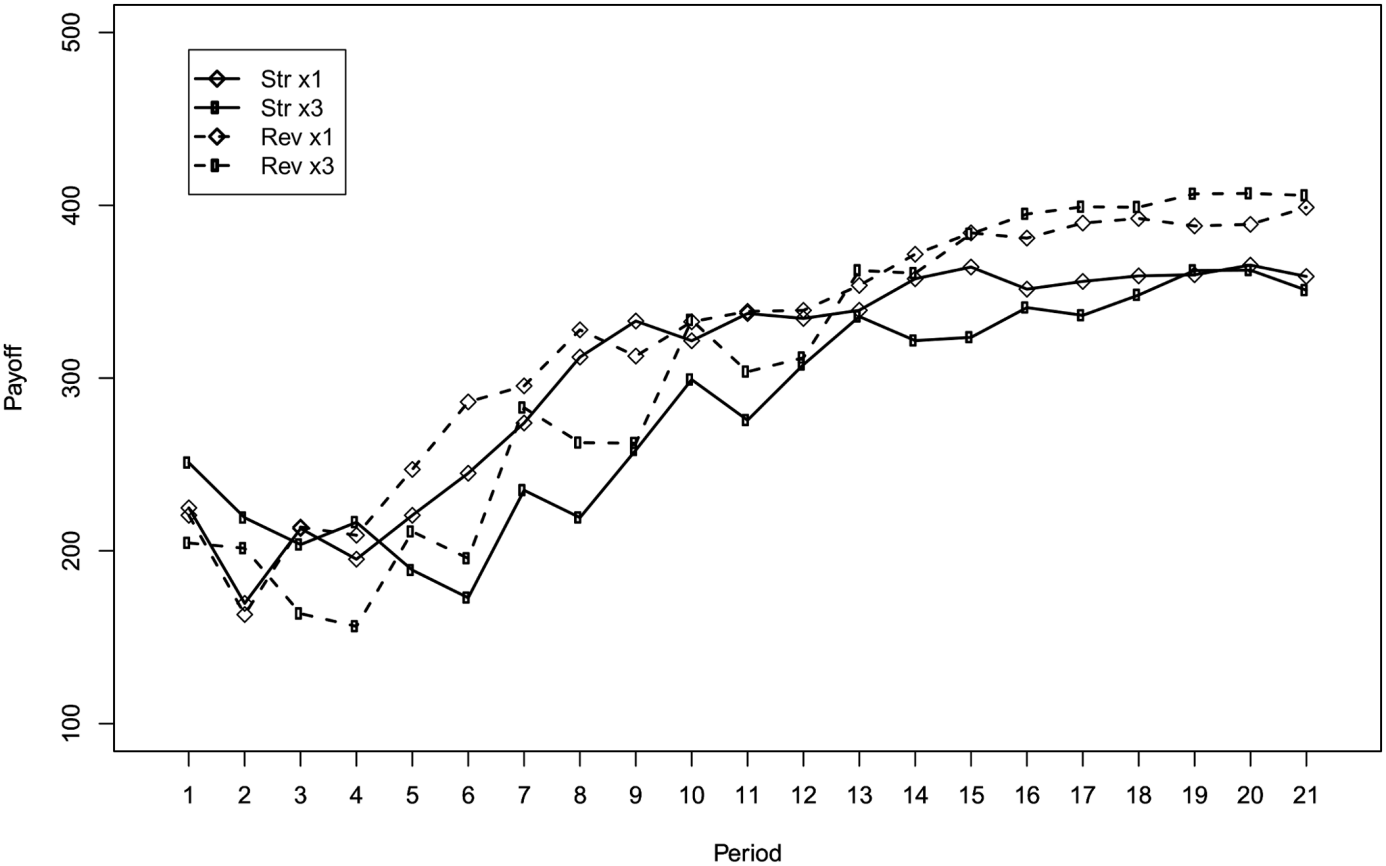
Average payoff by round and treatment.

Fig 3 shows the average payoff by round and treatment. As is apparent from the figure, the average payoff increases over rounds in all treatments and, in the final rounds, it reaches a higher level in the *reverse* compared to the *straight* treatments. The average payoffs over the 21 rounds reflects this pattern. Their mean is 304.4 ECU (SD = 108.2) and 282.3 ECU (SD = 106.1) in the *straight*-×1 and *straight*-×3 treatments and it is 320.7 ECU (SD = 147.9) and 305.2 ECU (SD = 115.2) in the *reverse*-×1 and *reverse*-×3 treatments.

**Fig 4:**
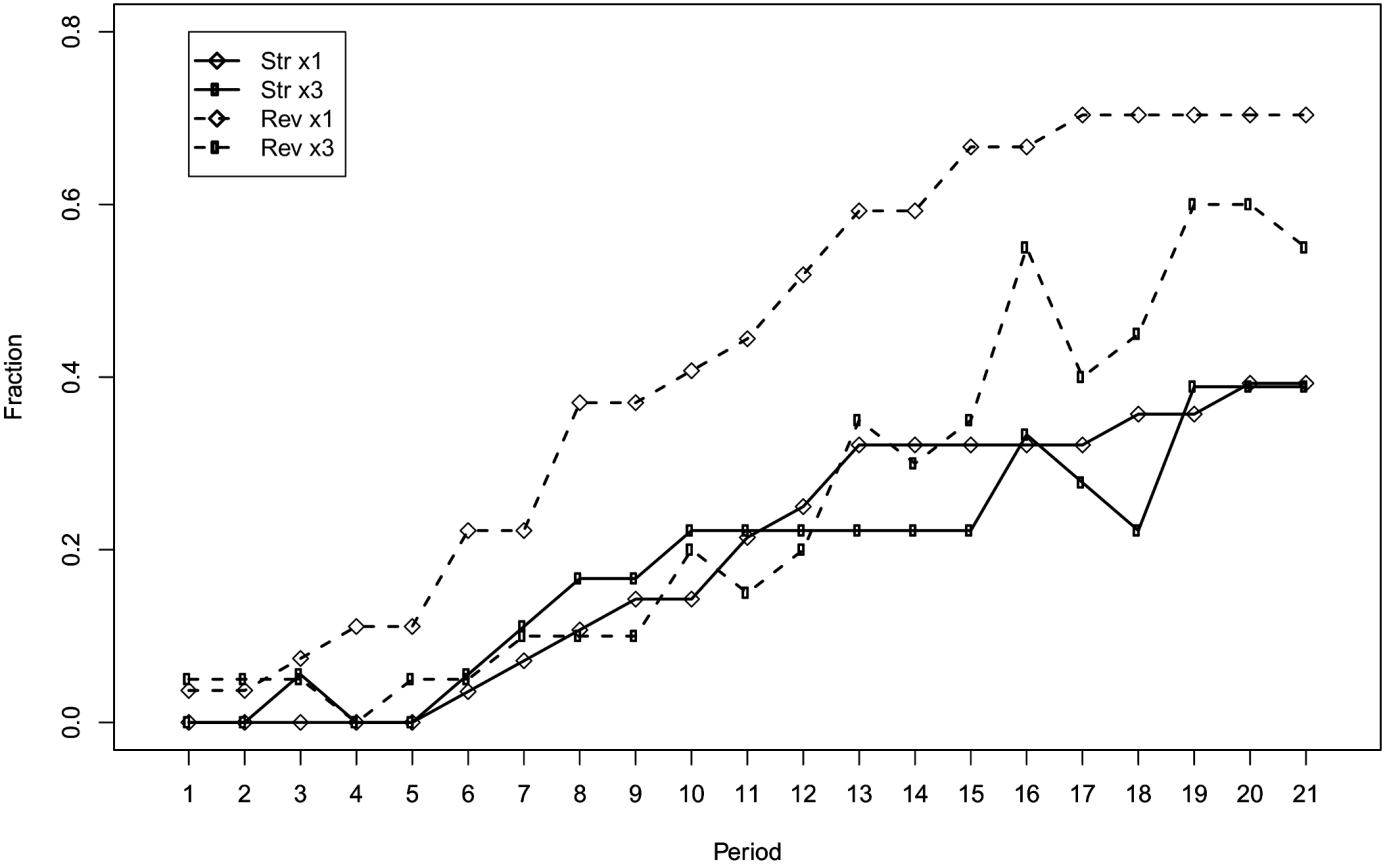
Fraction of efficient choices by round and treatment.

To have a clearer picture of the level of efficiency across treatments, we can look at Fig 4 which shows the fraction of subjects making efficient choices by round and treatment. From Fig 4 we can observe that the frequency of efficient subjects reaches higher levels in the *reverse* treatments compared to the *straight* ones. In the *reverse* treatments, 70% of the subjects reach efficiency in the final round when the frequency of feedback is ×1, while 55% of the subjects reach efficiency when the frequency of feedback is ×3. In the *straight* treatments, regardless of the frequency of feedback, only 39% of the subjects reach the efficient allocation in the final round. Both Figs 3 and 4 suggest that *framing* more than *frequency* of feedback drives the difference in performance among treatments.

To better identify which type of feedback is more effective at enhancing awareness of choice consequences and the search for better alternatives, we ran regression models (Table 2). Model 1 is a fractional response probit model with the fraction of rounds in which subjects are efficient as dependent variable, while Model 2 is a Cox proportional hazard model with the first round in which subjects reach efficiency as dependent variable. Both models share the same set of explanatory variables: (i) two treatment dummies, d(*reverse*) and d(×3), and their interaction d(×3) × d(*reverse*); (ii) a dummy variable controlling for gender d(female), (iii) the average mark obtained in the exams (demeaned), and (iv) the time in minutes spent in the laboratory to complete the real effort task.

**Table 2:** Regressions’ estimates with s.e. in parentheses.

	Mod. 1	Mod. 2
	FRM probit	Cox PH
	(robust se)	durat. mod.
(Intercept)	−0.6321**	—
	(0.2070)	—
d(×3)	−0.0368	0.1004
	(0.3028)	(0.4896)
d(*reverse*)	0.7203**	1.1078**
	(0.2535)	(0.3882)
d(×3) × d(*reverse*)	−0.5188	−0.6668
	(0.4353)	(0.6272)
d(female)	−0.3699^∘^	−0.6364*
	(0.2139)	(0.3112)
exam_mark	0.0141	0.0496
	(0.0353)	(0.0567)
time_effort_task	−0.0174	−0.0271
	(0.0118)	(0.0190)
*R*^2^	0.163	0.167
Concordance	—	0.669

Signif. codes:

‘***’ p-value ≤ 0.001

‘**’ 0.001 < p-value ≤ 0.01

‘*’ 0.01 < p-value ≤ 0.05

‘^∘^’ 0.05 < p-value ≤ 0.1

Starting with the results of the fraction of efficient choices in Model 1, we confirm the insight that a negative frame significantly fosters the search for an efficient allocation. In fact, the variable d(*reverse*) is positive and significant. This shows that the expected fraction of rounds in which subjects make efficient choices is higher in the *reverse* treatments than in the *straight* ones. As a second result, the lack of significance of the variable d(×3) suggests that the diluted frequency of feedback does not impact subjects’ performance. More precisely, the fraction of efficient choices when feedback about obtained payoff is given every three rounds does not differ from when it is given every round. This result shows that, in our task and over the 21 experimental periods, the continuous provision of feedback does not lead to better individual performances. Finally, looking at the estimate of the term d(×3) × d(*reverse*) we can exclude the presence of interaction effects between framing and frequency of feedback. Among the control variables we find a weakly significant effect of the gender dummy, suggesting that the fraction of efficient choices made by women is lower than that by men.

Moving to the results on the duration of the search before reaching the efficient allocation in Model 2, we observe that the estimated parameter of d(*reverse*) is positive and significant. This estimate implies that, in each round, the expected hazard of reaching the efficient allocation in the *reverse* treatment is 202% higher than the expected hazard in the *straight* one (the estimated parameter implies an expected hazard ratio of 3.02). In other words, subjects exposed to feedback including losses are significantly more likely to search for an alternative efficient allocation compared to those exposed to feedback including benefits. Model 2 confirms that the diluted frequency of feedback has no significant effect on the number of rounds needed to reach the efficient combination of allocation choices (d(×3) is not significant). Finally, Model 2 confirms the effect of gender on search of efficiency: women show a lower hazard of reaching the efficient allocation compared to men (47% lower).

### Social feedback

**Fig 5:**
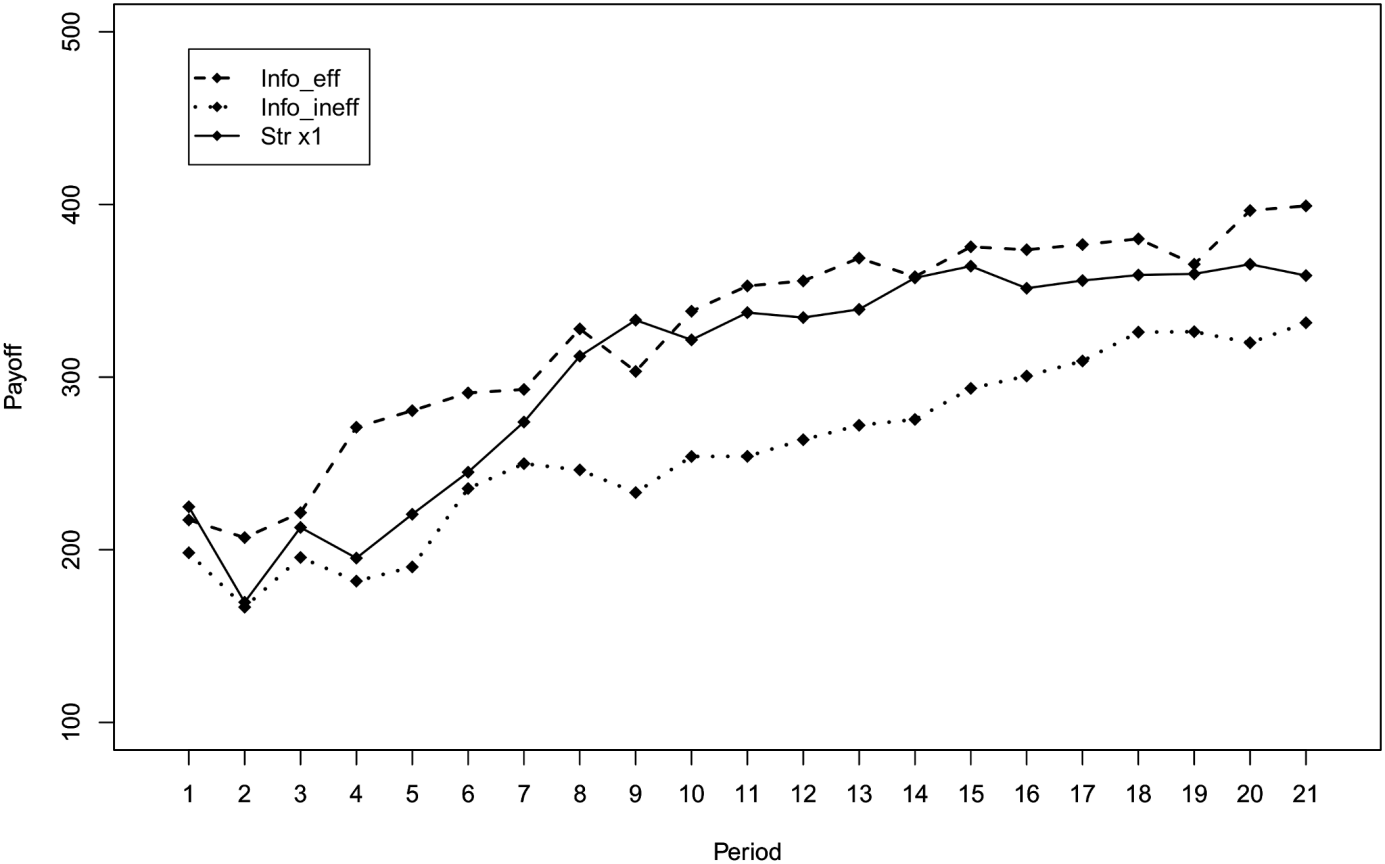
Average payoff by round and treatment.

In this subsection, we analyze the impact of social feedback on efficient behavior. Looking at Fig 5, which reports the average payoff by round and treatment, we can see that in the second half of the experiment payoffs are ranked as expected: the average payoff in the *info-eff* treatment is higher than that in the *straight*-×1 treatment that, in turn, is higher than that in the *info-ineff* treatment. Also, the means of the subject’s average payoff are ranked in the same way, with a mean of 258.3 ECU (SD = 79.7) in the *info-ineff* treatment, 304.4 ECU (SD = 108.2) in the *straight*-×1 treatment, and 326.3 ECU (SD = 108.1) in the *info-eff* treatment.

**Fig 6:**
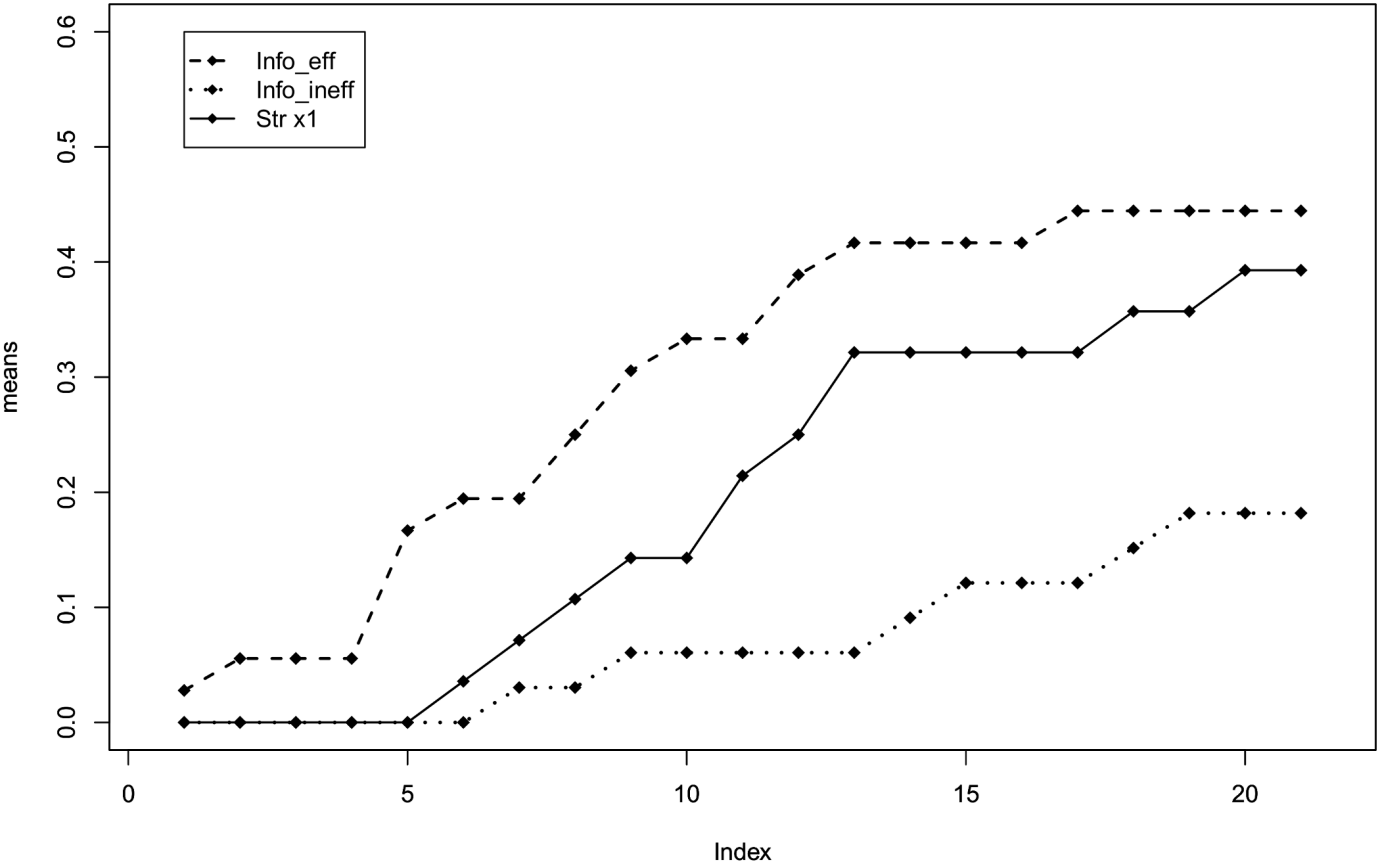
Fraction of efficient choices by round and treatment.

Fig 6 shows the fraction of efficient choices by round and treatment and provides similar insights into how feedback on the best performer’s obtained payoff affects behavior. We observe that the fraction of efficient choices in the last rounds is 39% in the *straight*-×1 and 44% in the *info-eff* treatments, while it is only 18% in the *info-ineff* treatment.

These results suggest that feedback about the best performer’s payoff represents an anchor for subjects that may reduce exploration and settle for a satisfying payoff level. Table 3 shows results from an econometric test of this insight. Model 3 and Model 4 follow the specifications of the two models reported in Table 2. More precisely, Model 3 is a fractional response probit model with the fraction of rounds in which subjects are efficient as dependent variable, and Model 4 is a Cox proportional hazard model with the first round in which subjects reach efficiency as dependent variable. As for the dependent variables, these models differ from Models 1 and 2 only for the treatments dummies. The variables d(*no info*) and d(*info ineff*) capture the effect of providing no information about the best performer’s obtained payoff and the effect of providing information about an inefficient best performer compared to the case of providing information about an efficient best performer.

**Table 3:** Regressions’ estimates with s.e. in parentheses.

	Mod. 3	Mod. 4
	FRM probit	Cox PH
	(robust se)	durat. mod.
(Intercept)	−0.2935	—
	(0.1853)	—
d(*no info*)	−0.2517	−0.3072
	(0.2500)	(0.3956)
d(*info ineff*)	−0.8097***	−1.1357*
	(0.2927)	(0.4893)
d(female)	−0.5981**	−0.8420*
	(0.2480)	(0.3949)
exam_mark	0.0230	0.0504
	(0.0443)	(0.0746)
time_effort_task	−0.0150	−0.0150
	(0.0095)	(0.0175)
*R*^2^	0.216	0.134
Concordance	—	0.696

Signif. codes:

‘***’ p-value ≤ 0.001

‘**’ 0.001 < p-value ≤ 0.01

‘*’ 0.01 < p-value ≤ 0.05

‘^∘^’ 0.05 < p-value ≤ 0.1

Looking at the fraction of efficient choices in Model 3, we find that subjects informed about the payoffs of an inefficient best performer show a significantly lower fraction of efficient choices compared to those informed about the payoffs of an efficient best performer (d(*info ineff*)). We also observe no significant difference in performance between subjects that do not receive information and subjects that receive information about the efficient best performer (d(*no info*)). These results show that the type of social content embedded in the information provided to the subjects is crucial for enhancing or inhibiting the search for better alternatives. In particular, we observe that more information is not always better than less. While receiving information on the payoff obtained by virtuous best-performers seems to have no impact on performance, receiving information about inefficient ones seems to have a detrimental effect on behavior. Among the control variables, the gender dummy is negative and weakly significant, confirming the effect observed in the other treatments.

Looking at the number of rounds necessary to reach the efficient allocation in Model 4, we observe that the estimated parameter of d(*info ineff*) is negative and significant. Therefore, the hazard of reaching an efficient allocation when receiving information about the inefficient best performer is lower than when receiving information about the efficient best performer (the expected hazard is 68% lower). As a second result, we find no significant difference in the hazard of reaching an efficient allocation when information on the obtained payoff is not provided compared to when information on the payoff obtained by the efficient best performer is provided. Overall, the results obtained with the duration analysis confirm the results observed when looking at the fraction of efficient choices: information on the payoff obtained by an inefficient best performer reduces the likelihood to explore, i.e., measured in terms of an increase in the number of rounds, the efficient allocation. As a final observation, we find a significant gender effect with women showing a lower hazard of reaching efficiency compared to men (57% lower).

## Discussion and conclusions

In this study, we investigated how different types of feedback enhance individuals’ awareness of choice consequences and search for efficient alternatives. In particular, we focused on the decision problem faced by an individual who has limited awareness of her choice consequences.

To isolate and quantify the effect of feedback on behavior, we ran a laboratory experiment. We exploited the laboratory setting to have internal control over the mechanisms underlying feedback effects in a complex decision context, such as the one in which individuals are not aware of their choice consequences.

We add to the methodology of the learning research field by introducing a novel task: in our setting individuals are endowed with a fixed amount of points that when allocated to five different items provide a monetary payoff. Subjects are asked to obtain the highest payoff they can by exploring alternative allocations of points. We introduced feedback as a mechanism to enhance awareness of choice consequences and to foster the search for better allocations. We varied feedback along three dimensions: framing, frequency, and social content. We created six treatments to isolate the effect of each of the three types of feedback on efficient behavior.

Our first finding relates to framing. When feedback contains obtained payoff in terms of losses, it elicits the highest effect on learning and fosters the search for efficient behavior. This pattern reflects the phenomenon of *loss aversion* [32] and *loss attention* [33]. When feedback includes losses associated with past choices, compared to when it includes benefits, individuals become significantly more sensitive to the consequences of their choices and more focused on the task. As a result, they are more likely to search for better options. This result provides important implications for policymakers. For instance, to promote efficient behavior, policymakers should leverage negative aspects, such as losses and costs, by means of informative devices.

The second finding concerns frequency. Diluting the frequency of feedback about allocation choices and obtained payoff does not elicit a significant behavioral change. This result may appear puzzling. Indeed, it is natural to expect worse performances when feedback is delivered less frequently. A possible explanation may be that 21 rounds are not enough for the difference to emerge. Although we cannot rule out this hypothesis, we must stress that both the average payoff and the percentage of efficient choices in the two frequency conditions tend to converge rather than diverge over rounds (see Figs 3 and 4). Additionally, we argue that observing a similar performance in the two frequency conditions can be explained by the design of the experiment. With the ×3 treatment, we wanted to test whether the frequency of feedback has an impact on behavior by keeping the amount of information constant. Hence, the difference between ×1 and ×3 is milder than it appears at a first glance: when a subject receives feedback in the ×3 treatment, she is informed about the points allocated to the sliders and the payoff she obtained in all the previous three rounds. As a consequence, the search for efficient allocations may be slower in the ×3 treatment but not significantly so. The fact that a small reduction of the frequency of feedback has little impact on performances has important policy implication in those cases where sending the feedback is costly. Indeed, instead of sending feedback after each decision, one can group feedback about multiple decisions without losing much in terms of performance.

Our third finding is with regard to social effects. We find that providing individuals with feedback containing social information on suboptimal peers is detrimental for eliciting changes toward efficient behavior. In particular, we find that providing information on the payoff obtained by an efficient best-performer has no effect on behavior. On the other hand, providing information on the payoff obtained by an inefficient best-performer significantly worsens the individual likelihood to search for better alternatives. In this latter scenario, individuals anchor their behavior to the benchmark suggested by feedback. Providing information about suboptimal peers leads individuals to satisfying behavior by lowering their aspiration levels. This result is also of practical relevance for policymaking. It is better to avoid providing individuals with information triggering social comparison unless the other to compare with is a virtuous example to imitate.

Fourth, across all treatments we find women to be less willing to search for better alternatives. This is in line with the widely documented gender difference in economic preferences [54]. One potential fruitful direction for future research would be to exploit the external validity of field settings to address gender difference in the willingness to search for better alternatives. As an example, we could vary the variance associated with each choice consequence by providing household women and men with tailored information leaflets about the energy usage of each individual appliance and test whether it cancelled out this gender difference.

## Supporting information

S1 File Contains robustness check analysis and experimental instructions. (PDF)
