# Supplementary material for: Feedback and efficient behavior
Source: PLoS One. 2017 Apr 21;12(4):e0175738. doi: 10.1371/journal.pone.0175738 (PMC5400271; doi:10.1371/journal.pone.0175738)
Supplement: S1 File — (PDF) [file pone.0175738.s001.pdf]

S1 Supporting Information

Robustness check

Table A reports the results of two random effect censored regression models where payoffs are used as dependent variable. These models allow us to control for both the within-subject correlation and the censoring of payoffs at 500 ECU. Model 5 complements the analysis on the effect of framing and frequency reported in Table 2, and Model 6 complements the analysis on the effect of social feedback reported in Table 3.

The results of Model 5 show the same qualitative pattern obtained with the fractional response model and with the duration model (Mod. 1 and Mod. 2), but the effect of framing fails to reach the significant level. Model 6, instead, perfectly replicates the results obtained with the fractional response model and with the duration model (Mod. 3 and Mod. 4).

Table A. Random effect Tobit (dep. var. Payoff, upper limit 500 ECU)

	Mod. 5 (se)	Mod. 6 (se)
(Intercept)	373.189***	396.474***
	(33.612)	(22.154)
d(×3)	-20.580	—
	(46.254)	—
d(*reverse*)	51.860	—
	(41.119)	—
d(×3)×d(*reverse*)	-20.930	—
	(65.102)	—
d(*no info*)	—	-32.491
	—	(30.004)
d(*info ineff*)	—	-80.478**
	—	(28.839)
d(female)	-51.745	-69.657**
	(32.935)	(24.516)
exam_mark	0.628	2.642
	(5.821)	(5.112)
time_effort_task	-5.589**	-2.283**
	(2.093)	(1.059)
σ_u	145.892***	113.963***
	(11.589)	(8.859)
σ_e	147.572***	119.963***
	(2.980)	(2.209)
ρ	0.494	0.474
	(0.040)	(0.039)
Loglik	-9637.15	-10654.89
n groups	93	97
uncensored observations	1422	1648
right-censored observations	531	389

Signif. codes:
‘***’ p-value ≤ 0.001
‘**’ $0.001 <$ p-value ≤ 0.01
‘*’ $0.01 <$ p-value ≤ 0.05
‘◦’ $0.05 <$ p-value ≤ 0.1

Experimental Instructions

These are translated versions (originally in Italian) of the instructions used in the experiment. The instructions changed accordingly to the treatment, differences are indicated in the text.

Thank you for taking part in this experiment. Please read carefully these instructions: a certain amount of money will be paid as a result of decisions made in the experiment: your earning will be paid to you at the end of the experiment. In any case, a show-up fee of €4 will be paid for taking part to this experiment.

The experiment is made of two phases which are conducted in two days (Phase 1 today, Phase 2 tomorrow): your payment will be made at the end of Phase 2. This means that if tomorrow you will not show up for Phase 2, you will not be paid (not even the show-up fee of €4).

During the experiment, you are not allowed to talk to other participants. Please also turn off your mobile phone. Violations of these rules will lead to your exclusion from the experiment and all payments. Whenever you have a question, please raise your hand and an experimenter will come to answer your question in private.

Please note that your decisions are anonymous in the sense that other participants will not be able to link them to your identity. The data generated will only be used for scientific purposes.

Phase 1

The following instructions only refer to Phase 1. Instructions for Phase 2 will be provided tomorrow.

In Phase 1 you are asked to count the number of 0 in different tables. For each solved table, you will earn 50 experimental points. Your goal is to collect 1050 experimental points by solving 21 tables. You have 50 minutes for completing the task. If you do not solve 21 tables in the provided time (or if you leave the experiment before completing the task), you will not allowed to take part in Phase 2 and you will excluded by any payments.

The experimental points that you earn today may be exchanged, in Phase 2, into Euro according to the decisions you will take tomorrow.

Phase 2

Welcome back!

By completing Phase 1, you earned the right to participate in Phase 2. In Phase 2 you have to allocate the experimental points you earned yesterday. More precisely, Phase 2 is composed by 21 rounds: in each round you have to decide how to allocate 50 experimental points among 5 different sliders.

Every slider convert points into Experimental Currency Unit (ECU): you can allocate to each slider up to 20 points. These ECUs represent [for treatments straight- only: **earnings**] [for treatments reverse- only: **costs**] and your goal is to [for treatments straight- only: **maximize**] [for treatments reverse- only: **minimize**] the amount in ECU of each slider by identifying the number of points that gives the [for treatments straight- only: **maximum**] [for treatments reverse- only: **minimum**] amount.

The total [for treatments straight- only: **earning**] [for treatments reverse- only: **cost**] of the round is computed as the sum of the results of each slider: the [for treatments straight- only: **maximum total earning**]

for treatments reverse- only: **minimum total cost** (which is reachable by distributing 50 points) is equal to for treatments straight- only: **500 ECU** for treatments reverse- only: **0 ECU**.

You will start each round with an initial endowment of to for treatments straight- only: **0 ECU** for treatments reverse- only: **500 ECU** to which will be for treatments straight- only: **added the total earning** for treatments reverse- only: **subtracted the total cost** for determining the final payoff of the round.

You will get feedback on the result of the round for treatments -x1 only: **at the end of each round.**

for treatments -x3 only: **every three rounds (together with the feedbacks on the previous round), thus after round 3, 6, 9, 12, 15, 18 and 21.**

for treatments info- only: **In addition you will get also the information on the result of the best performer who took part in your same task in a previous section.**

At the end of Phase 2, one round will be randomly selected for your final payment: the final payoff of the selected round will be converted into euro according to the exchange rate 25 ECU = 1€. Before leaving the room, after completing a short questionnaire, the amount converted in euro plus the show up fee will be privately paid to you.
